# Composition of soil *Frankia* assemblages across ecological drivers parallels that of nodule assemblages in *Alnus incana* ssp. *tenuifolia* in interior Alaska

**DOI:** 10.1002/ece3.11458

**Published:** 2024-07-08

**Authors:** M. D. Anderson, D. L. Taylor, K. Olson, R. W. Ruess

**Affiliations:** ^1^ Biology Department Macalester College Saint Paul Minnesota USA; ^2^ Institute of Arctic Biology University of Alaska Fairbanks Alaska USA; ^3^ Department of Biology University of New Mexico Albuquerque New Mexico USA

**Keywords:** *Alnus*, *Frankia*, mutualism, nitrogen fixation, plant–microbe interactions

## Abstract

In root nodule symbioses (RNS) between nitrogen (N)‐fixing bacteria and plants, bacterial symbionts cycle between nodule‐inhabiting and soil‐inhabiting niches that exert differential selection pressures on bacterial traits. Little is known about how the resulting evolutionary tension between host plants and symbiotic bacteria structures naturally occurring bacterial assemblages in soils. We used DNA cloning to examine soil‐dwelling assemblages of the actinorhizal symbiont *Frankia* in sites with long‐term stable assemblages in *Alnus incana* ssp. *tenuifolia* nodules. We compared: (1) phylogenetic diversity of *Frankia* in soil versus nodules, (2) change in *Frankia* assemblages in soil versus nodules in response to environmental variation: both across succession, and in response to long‐term fertilization with N and phosphorus, and (3) soil assemblages in the presence and absence of host plants. Phylogenetic diversity was much greater in soil‐dwelling than nodule‐dwelling assemblages and fell into two large clades not previously observed. The presence of host plants was associated with enhanced representation of genotypes specific to *A. tenuifolia*, and decreased representation of genotypes specific to a second *Alnus* species. The relative proportion of symbiotic sequence groups across a primary chronosequence was similar in both soil and nodule assemblages. Contrary to expectations, both N and P enhanced symbiotic genotypes relative to non‐symbiotic ones. Our results provide a rare set of field observations against which predictions from theoretical and experimental work in the evolutionary ecology of RNS can be compared.

## Graphical abstract

N‐fixing host plants can theoretically affect the structure of soil‐dwelling assemblages of symbiotic microbes via selective amplification of specific genotypes. However, little is known about how soil‐dwelling and nodule‐dwelling assemblages compare in natural populations. This study examined this comparison in the Alnus‐Frankia system across a primary successional gradient in interior Alaska.

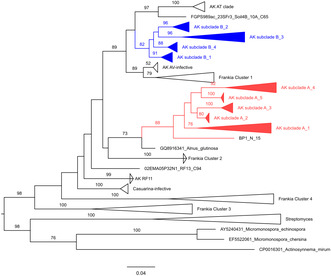

## 1 INTRODUCTION

Root nodule symbioses (RNS) between plants and nitrogen (N)‐fixing bacteria are ecologically and economically important interactions (Graham & Vance, 2003; Vitousek et al., 2002; Wheeler & Miller, 1990), and provide model systems for studying the evolutionary ecology of mutualism (Heath & Grillo, 2016). Three major groups of RNS exist: (1) “rhizobial” symbioses that occur between three proteobacterial phyla and plant hosts from the family Fabaceae and the genus *Parasponia*, (2) “actinorhizal” symbioses between 25 plant genera and the single actinomycete genus *Frankia*, and (3) associations between cyanobacteria and some cycad species (Dawson, 2008; Piex et al., 2015; Vessey et al., 2005). In all RNS, host plants acquire bacterial symbionts “horizontally”; that is, plants form nodules de novo with bacteria found in the soil environment, rather than receiving symbionts “vertically” from their parents, as in some endosymbiotic interactions (Denison & Kiers, 2011; Frank, 1996; Wall & Berry, 2008). The independent existence of plant and bacterial partners in RNS affects the evolution of the symbiotic interaction in several ways.

One result of this independence is the potential for evolutionary conflict between host plants and symbiotic bacteria. Individual plants associate simultaneously with bacteria from multiple lineages that can vary widely in the benefits they provide to the host (Denison, 2000; Kiers & Denison, 2008; Markham, 2008; Parker, 1995; Sachs et al., 2010). This can create a tragedy of the commons in bacterial communities (Denison, 2000), favoring potentially “selfish” behavior such as decreased allocation of host‐derived carbon to N‐fixation (Oono & Denison, 2010), or unregulated reproduction of bacteria within host nodules (Cotin‐Galvan et al., 2016). Over evolutionary time, selection for such “cheating” behavior should destabilize the mutualism, in the absence of other factors (Sachs & Simms, 2006). However, bacterial cheating is thought to be held in check by several countermeasures evolved by host plants. A multi‐level system of pre‐nodulation communication mechanisms (Clúa et al., 2018) between roots and bacteria allows the plant to recognize bacterial genotypes likely to provide effective N‐fixation (“partner choice”; Kiers & Denison, 2008). Post‐nodulation detection of bacterial N fixation allows plants to “punish” cheating genotypes via decreased reproduction within nodules (termed “host sanctions” (Denison, 2000)) and/or selectively allocate resources to especially beneficial bacterial genotypes (“differential rewards”; Simms & Taylor, 2002).

A second result of horizontal transmission is that the bacterial partner occupies two distinct niches: a free‐living existence in soil, and a state in which they essentially act as plant organelles. In symbiotic mode, bacteria fix N using host‐derived carbon that, in the most effective symbiotic genotypes, is allocated preferentially to N fixation over bacterial reproduction (Benson & Silvester, 1993; Denison & Kiers, 2004). These two niches are likely to exert different selection pressures on N‐fixing bacteria, which may result in tradeoffs between symbiotic and free‐living lifestyles (West et al., 2002) and/or drive repeated gains and losses of symbiotic capability in bacterial lineages (Sachs et al., 2010; Sachs & Simms, 2006).

Little is known about the relationship between nodule and soil assemblages of N‐fixing bacteria in natural environments. Historically, investigation of diversity patterns in soil‐dwelling bacterial symbionts has been based on the use of N‐fixing “trap plants” to sample bacterial communities via nodule formation or, less frequently, on direct isolation of bacteria from soils (Chaia et al., 2010; McInnes et al., 2004). Both approaches are subject to significant and well‐known biases (Chaia et al., 2010; Kirk et al., 2004). In recent years, molecular methods such as DNA cloning, qPCR, and next‐generation sequencing (NGS) methods have been increasingly used to characterize soil communities of N‐fixing bacteria (e.g., Ben Tekaya et al., 2017, 2018; Miranda‐Sánchez et al., 2016; Rodriguez et al., 2016). While these methods are not without their own biases (Acinas et al., 2005; Chandler et al., 1997; Gołębiewski & Tretyn, 2020; Sáenz et al., 2019; Sanchez‐Cid et al., 2022; van Elsas & Boersma, 2011), they allow investigation of a much broader swath of the bacterial community not limited to genotypes able to induce host nodules and/or grow under laboratory conditions. In the present study, we use PCR and DNA cloning to examine patterns of genetic diversity in naturally occurring soil‐dwelling populations of the actinorhizal symbiont *Frankia* spp. associated with the host species *Alnus incana* ssp. *tenuifolia* (hereafter “*A. tenuifolia*”) across a primary successional sere in the boreal forest of Alaska. We utilize a subset of research sites in which we have previously observed stable nodule‐dwelling assemblages over a range of 3–5 years, with large differences in composition among successional stages that are consistent among replicate sites (Figures S1 and S2) (Anderson et al., 2009, 2013; Ruess et al., 2013). In the present study, we investigated three questions: (1) how do soil and nodule assemblages compare in phylogenetic diversity of *Frankia*; (2) do soil and nodule assemblages change in parallel in response to environmental variation: both across succession and in response to long‐term fertilization with nitrogen (N) and phosphorus (P); and (3) do soil assemblages differ in the presence and absence of *A. tenuifolia* individuals?

The actinobacterial genus *Frankia* is the microsymbiont involved in actinorhizal RNS. *Frankia* forms effective root nodules with plants from eight families and 25 genera within the eurosid I clade of flowering plants (Pawlowski & Demchenko, 2012; Swensen & Benson, 2008). Phylogenetic studies of *Frankia* using both single‐ and multi‐locus approaches robustly indicate that the genus is divided into four clusters differing in host genus specificity. Cluster 1 *Frankia* form root nodules with alders (*Alnus* spp.) as well as *Morella* spp., *Casuarina* spp., and *Comptonia* spp. (Swensen & Benson, 2008). *Alnus*‐infective *Frankia* are phylogenetically and functionally diverse. Within *Frankia* cluster 1, genotypes have been described that differ in ability to form nodules on specific alder species (Markham, 2008; Vemulapally, Guerra, & Hahn, 2022; Vemulapally, Guerra, Weckerly, et al., 2022), to sporulate within host nodules (Cotin‐Galvan et al., 2016; Markham, 2008; Pozzi et al., 2015), to subsist on specific carbon sources in soil such as leaf litter (Mirza, Welsh, & Hahn, 2009), and to support the growth and N fixation of specific alder species (Markham, 2008; Prat, 1989; Sellstedt et al., 1986). Alder species appear to exert considerable control over the symbiosis. Surveys of nodules in natural habitats indicate genetic differences in symbiotic *Frankia* among alder species, even when they occur in the same sampling site (Anderson et al., 2009; Pokharel et al., 2011), and bioassay studies have demonstrated the ability of different species to associate with different *Frankia* genotypes from the same soil (Lipus & Kennedy, 2011). Recent experiments comparing soil‐ and nodule‐dwelling *Frankia* using microcosms in which the concentration of specific *Frankia* genotypes was controlled demonstrate that alder species can selectively associate with *Frankia* genotypes disproportionately to their relative occurrence in soil and that the proportion of selected types differs across some alder species (Ben Tekaya et al., 2018; Vemulapally, Guerra, & Hahn, 2022; Vemulapally, Guerra, Weckerly, et al., 2022).

In ecosystems of the Tanana River floodplain, *A. tenuifolia* demonstrates both genetic and environmental specificity in its association with *Frankia*. >95% of the nodules we have collected in several prior studies contain *Frankia* belonging to a single clade (the “AT clade”) that clusters within the larger *Alnus*‐infective group but is divergent from other *Frankia* groups within it (Anderson et al., 2009, 2013; Ruess et al., 2013). This specificity appears to be a function of the host plant: in sites that contained a second species of *Alnus*, *A. viridis*, both host species harbored phylogenetically distinct *Frankia* despite occurring in close proximity to one another (Anderson et al., 2009). Within the *A. tenuifolia*‐associated clade, individual genotypes are distributed non‐randomly across successional environments: nodules in early‐succession monospecific alder stands almost exclusively contain a single genotype (“RF7”) not found in later‐succession ecosystems; nodules from mid‐succession forests dominated by balsam poplar mostly contain a nearly even mix of two other genotypes (“RF1” and “RF3”); and nodules from late‐succession white spruce forests contain a decreased proportion of the two mid‐succession types, as well as a higher richness and evenness of other genotypes (Anderson et al., 2009, 2013; Ruess et al., 2013). The association of specific genotypes with successional stages is robust: we have observed the same pattern in replicated, spatially intermixed sites representing each stage, and in repeated samples taken several years apart in a given site (Figures S1 and S2).

## 2 MATERIALS AND METHODS

### 2.1 Location and study system

This study was conducted on the Tanana River floodplain in the Bonanza Creek Experimental Forest (BCEF), a Long‐Term Ecological Research (LTER) site in the northern boreal forest near Fairbanks, Alaska. Details of the climate and ecosystem have been published elsewhere (Anderson et al., 2009; Ruess et al., 2013). Our study system is the actinorhizal RNS that occurs between thinleaf alder (*Alnus tenuifolia*) and bacteria of the genus *Frankia*. *A. tenuifolia* is an abundant and ecologically important woody shrub throughout interior Alaskan floodplain ecosystems, in which its life history and ecological impacts have been well studied. On the Tanana River floodplain, *A. tenuifolia* colonizes alluvial deposits within a few years following deposition, forming very dense stands (~10,000 stems/ha) that provide significant quantities of organic matter and N to early primary successional ecosystems (Hollingsworth et al., 2010; Van Cleve et al., 1993). In later stages of succession dominated by overstory balsam poplar (*Populus balsamifera*), and later, white spruce (*Picea glauca*), *A. tenuifolia* persists in the understory, occasionally proliferating to moderate density in canopy gaps (Hollingsworth et al., 2010).

Sites in the present study include one early‐succession alder site (FPE3), one late‐succession site dominated by white spruce (FPL2) examined in Anderson et al. (2009, 2013), and one mid‐succession balsam poplar‐dominated site (BP1) described in Ruess et al. (2013). The early‐ and late‐succession sites were selected to differ maximally in composition of *Frankia* in alder nodules, based on prior observations, in order to maximize our chances of detecting differences in soil assemblages. The mid‐succession site is part of a long‐term field fertilization study which includes plots fertilized with N (100 kg N*ha^−1^*year^−1^ as NH_4_NO_3_, “+N”) or P (80 kg P*ha^−1^*year^−1^ as P_2_O_5_, “+P”) for ≥5 years, and unfertilized control (“CTL”) plots (details in Ruess et al., 2013). Soil samples were collected from early‐ and late‐succession sites in July 2008, and from mid‐succession sites in August 2009.

### 2.2 Sample collection and processing

In early‐ and late‐succession sites, six individual *A. tenuifolia* plants were haphazardly selected for sampling. At each plant, a 1 × 1 m plot was established with the plant at the center. Within each quadrant of this plot, two soil cores (3 × 15 cm) were collected from randomly selected locations, for a total of eight cores from each plant. In the late‐succession site, an additional six 1 × 1 m areas of open forest floor not occupied by *A. tenuifolia* were also sampled in the same way to examine the effect of alder presence versus absence on the structure of *Frankia* assemblages. In the lab, all nodules and roots were removed and soil from each core was homogenized through a 1 mm sieve. Sieved soil was then lyophilized to constant weight and ball milled on a vortexer. Milled soils were pooled by successional stage and, in late succession, by presence or absence of alder, for a total of three soil sources (early, late alder, and late non‐alder). Pooling was achieved by combining 1.000 g samples from each core and homogenizing on a roller mill for ≥24 h. In mid‐succession sites, 10 soil cores (2.5 cm × 15 cm) were collected from random locations within each 20 m × 20 m plot (CTL, +N, and +P), and pooled using the same method as for the soil cores from the other sites, for a total of one soil source per treatment. In short, our field collection methods were designed to achieve a representative sample for each treatment (CTL, +N, and +P) or collection source (early‐succession, late‐succession alder, or late‐succession non‐alder) by capturing the variation within and among plants/locations in each condition (treatment/source), then pooling all field replicates equally into a single sample per condition. However, because this yields only a single replicate per condition for lab analysis, variation within each condition cannot be estimated.

### 2.3 DNA extraction, PCR, cloning, and sequencing

Genomic DNA was extracted from a maximum of 1.0 g of soil using the MoBio Powermax kit following manufacturer's instructions. PCR of soil DNA extracts utilized Redtaq (0.25 units/rxn) (Sigma‐Aldrich Corp., St. Louis, MO), and primers FGPS989ac (GGGGTCCGTAAGGGTC) (Bosco et al., 1992) and 23SFr3‐ten (GGCAWGGGTGACAGGATTTA), the latter a degenerate version of a primer we developed for this study. The forward primer has 100% sequence homology to *Frankia* strain ACN14a, the genomic representative for the *Alnus*‐infective *Frankia* clade (Normand et al., 2007). One component of the degenerate reverse primer is also a 100% match to this strain; the other component differs at a single location to target an SNP unique to the AT clade (Anderson et al., unpublished data). This primer set targets the non‐coding intergenic spacer region (IGS) between the 16S and 23S ribosomal RNA loci. Cycling conditions were as follows: initial denaturation at 94°C for 4 min, 35 cycles of 94°C for 1 min, annealing for 0.30 s (55°C for FGPS989ac/23SFr3‐ten), 72°C for 1.30 min, and finally, 72°C for 5 min. Amplicons from soil DNA extracts were cloned into the TOPO TA 4.0 vector with chemically competent top 10 cells following the manufacturer's instructions (Invitrogen, Carlsbad, CA). Individual colonies (100 per library) were selected at random from each plate for PCR amplification using M13 forward and reverse primers, and sequencing of the insert, which was performed at Functional Biosciences (Madison, WI). We opted to clone our PCR products because the high GC content of the target locus interferes with sequencing. We were able to obtain much cleaner reads using the M13 vector primers. We hypothesize that the cloning and Sanger sequencing approach used here should result in less bias (particularly against high GC targets) than a direct NGS approach, and also provide longer contiguous sequences for analysis than the NGS methods available at the time of our benchwork.

DNA sequences were edited using CodonCode Aligner (CodonCode Corporation, Centerville, MA), and multiple‐sequence alignments were generated using the Muscle (Edgar, 2004) and Mafft (Katoh et al., 2002) programs on the CIPRES (Miller et al., 2010) network. Sequence alignments were corrected by eye in Seaview (Gouy et al., 2010) and trimmed to common length using Bioedit (Hall, 1999). Likely chimeric sequences were identified using the program UCHIME (Edgar et al., 2011) as implemented in the mothur package (Schloss et al., 2009), and removed prior to subsequent analysis. Sequences derived from our mid‐succession control plots were consistently shorter than those from any other clone library. We, therefore, generated two alignments for analysis: one that spanned the entire rIGS but excluded mid‐succession control sequences (811 bp final alignment), and one that was truncated within the rIGS region and that only included sequences from mid‐succession libraries (627 bp final alignment). We used the former as the basis for our phylogenetic analysis and comparison of early‐ and late‐succession libraries and, within the latter, of alder and non‐alder soils. The mid‐succession‐only alignment was used to compare differences among fertilization treatments in our mid‐succession sites.

### 2.4 Phylogenetic analysis

Maximum‐likelihood (ML) phylogenetic analysis was performed using RAxML, version 8 (Stamatakis, 2014), as implemented on the CIPRES network, version 3.3 (Miller et al., 2010). We used a GTR + G substitution model and 1000 rapid bootstrap replicates. To facilitate comparison of our soil‐derived sequences with nodule‐dwelling *Frankia* from the same field locations, we sequenced the rIGS locus from 57 previously collected nodules representing the 13 most commonly observed genotypes across both *A. tenuifolia* and *A. viridis* host species (Anderson et al., 2009, 2013) that were based on restriction fragment analysis of the nifD‐K spacer locus. In order to place our sequences within a broader phylogenetic context, we also included reference sequences from Ghodbane‐Gtari et al. (2010), and a set of actinobacterial outgroups downloaded from GenBank.

Operational taxonomic units (OTUs) were defined in two ways: (1) based on well‐supported clades in the phylogenetic analyses, and (2) at specific levels of sequence similarity. For the former, clades were selected by eye based on distance (a selected clade had a long stem branch, relative to other clades), cohesion (a selected clade should lack long branches within the clade), and statistical support (≥70% bootstrap). Similarity threshold‐based OTUs were generated using the average neighbor clustering algorithm implemented in the “cluster” command in mother, version 1.0.0 (Schloss et al., 2009).

To check for sensitivity of our phylogenetic and OTU designation results to variation in our alignment, we used the program Gblocks (Castresana, 2000) to remove portions of the alignment deemed unreliable according to the most stringent, the least stringent, and an intermediate set of parameters. The resulting alignments were then analyzed using RAxML, and the best tree was qualitatively compared with the best tree based on the entire alignment.

### 2.5 Statistical analysis

Proportional representation of OTUs across experimental groups was calculated by means of two‐way contingency tables for each comparison of interest. Because DNA sequences derived from mid‐succession control soils were systematically shorter than sequences from all other groups, these soils were excluded from our cross‐succession comparison. This left us with four comparisons of interest: (1) early versus late succession, (2) alder versus non‐alder soils in late succession, (3 and 4) mid‐succession control versus mid‐succession fertilized soils (both N and P). Each comparison was made using a separate contingency table, with statistical significance of each bivariate relationship determined via likelihood ratio tests using the “contingency analysis” platform in JMP Pro v. 16.0. Statistical comparison of proportional differences in OTU representation across comparison groups was conducted using the “categorical analysis” platform in JMP Pro v. 16.0. Briefly, this platform allows a user to define a set of pair‐wise comparisons between levels of a categorical explanatory variable. Differences in levels of the categorical response variable are calculated, and statistical significance is determined, via likelihood ratio, Pearson chi‐square, and Fisher's exact tests (JMP, 2021). We based our comparisons on the latter, as it consistently provided the most conservative results.

## 3 RESULTS

### 3.1 Phylogenetics

**FIGURE 1:**
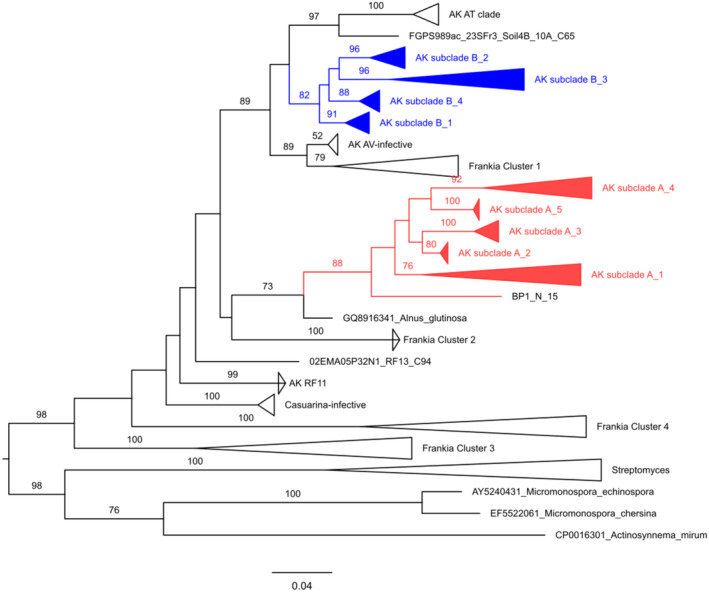
Maximum‐likelihood (RAxML) phylogeny of *Frankia* soil clones and reference sequences, and selected actinobacterial outgroups, based on rIGS locus. ML analysis was conducted on an alignment containing 811 positions across sequences from 559 *E. coli* clones containing 16S‐23S ribosomal intergenic spacer (rIGS) loci amplified from *Frankia* found in six Alaskan soils. Reference sequences for phylogenetic comparison included 57 rIGS sequences derived from previously collected nodules in each Alaskan study site, 58 publicly available sequences from across the *Frankia* genus (Ghodbane‐Gtari et al., 2010), and 10 sequences from outgroup taxa within the Actinomycetes. Branch labels represent percent bootstrap support, out of 1000 replicates. For clarity, only branches with ≥70% support are shown, except in special cases. Collapsed clades are labeled according to canonical *Frankia* cluster, in the case of comparison sequences, with letter_number indicators for sequences found only in soils in the present study, or according to groupings previously observed in nodules (“AK AT clade” in *A. tenuifolia* nodules and “AK AV‐infective” in *A. viridis* nodules). Single branch labels containing “RF” represent previously collected *A. tenuifolia* nodules; labels containing “soil” or “BP1” are single clones from the present study. Sequences from our Clade A are shown in red and Clade B in blue.

Sequence diversity in our soil samples was high: 559 soil clones yielded 393 unique sequences. ML analysis recovered the four canonical *Frankia* “Clusters” that are used as working taxonomic groups based on phylogenetic patterns recovered across multiple loci (Figure 1, Figure S3) (Normand et al., 1996; Nouioui et al., 2011; Pozzi, Bautista‐Guerrero, et al., 2018; Swensen & Benson, 2008), including Ghodbane‐Gtari et al. (2010), the source for our reference IGS alignment. All four *Frankia* clusters were well supported statistically (>75% bootstrap) in our tree; however, Cluster 3 rather than Cluster 4 was basal within *Frankia*, and Cluster 2 fell within the large group that contained both reference sequences derived from *Alnus* nodules and our soil clones. The position of Cluster 2 reflects the strongest ambiguity in our tree, as it differs from the standard position of these reference sequences, falling within rather than adjacent to a larger *Alnus*‐infective/*Casuarina*‐infective cluster that is broadly accepted.

All of our soil‐derived sequences fell into four strongly supported clades that were well within the group of previously described *Frankia* at the genus level (Figure 1, Figure S3). However, within the genus, none of our sequences were closely related to any of the *Frankia* sequences in our reference alignment (Figure 1, Figure S3). Only a small minority of our sequences (37 sequences, 6.6%) clustered near previously described *Alnus*‐infective *Frankia* (“Cluster 1”), forming a poorly supported group (BS = 52%) with well‐supported affinity for the canonical “Cluster 1” *Frankia* (BS = 89%). This small group included nodules from *A. viridis*, which contain genotypes of *Frankia* we have previously observed to have high specificity for this host species (Anderson et al., 2009). The majority of our soil‐derived sequences (374 sequences, 67.0%) fell into two well‐supported clades that formed a larger group with the “typical” *Alnus*‐infective strains, but were clearly differentiated from them. One clade, which we call the “AT clade” (BS = 100), includes nearly all the sequence variation we have observed in *A. tenuifolia* nodules in our Alaskan field sites. This group includes 162 soil‐derived sequences, or 29.0% of the total from this study. A second clade, which we call “Clade B”, is sister to this group (although this placement is not statistically supported), and only contains soil‐derived sequences observed in this study (211 sequences, 37.7%). A large portion of our sequences (148 sequences, 26.5%) form a unique fourth clade we call “Clade A”, that fell into the ambiguous portion of our tree, near previously described divergent sequences we have observed occasionally in our prior studies (unpublished data).

Sequences from the mid‐succession‐only alignment displayed a broadly similar phylogenetic pattern (Figures S4 and S5). Most soil‐derived sequences (128 sequences, 59.3%) fell into the AT clade with nodule‐forming reference sequences. The rest formed two clades, one sister to the AT clade, which we called “clade MID_1,” using numeric rather than letter designation to emphasize the different derivation of the mid‐succession sequences in our method, and one large clade that we split into two smaller groups: “MID_2” and “MID_3.” A total of 165 sequences were shared between the large rIGS and mid‐succession‐only alignments. Comparison of the clade assignments of these sequences in the two alignments indicated that mid‐succession clades MID_2 and MID_3 are equivalent to clade A from the larger alignment, and MID_1 is equivalent to clade B.

Both our clades A and B contained several well‐supported sub‐clades (Figure S3). The AT clade also contained three discernable sub‐groups: a basal group (“RF1_2_16”) composed of genotypes typical of late‐succession nodules (9 clones, 1.6%), a derived sub‐clade (“RF1_2_3_14”) that included most of the sequence types we have found in nodules of this host species (126 clones, 22.5%), and a sub‐clade (“RF7”) within this that includes sequences only found in early‐succession nodules of *A. tenuifolia* (27 clones, 4.8%). All three sequence groups have been previously observed to have high specificity for *A. tenuifolia* hosts in our sites (Anderson et al., 2009). Our sub‐clade designations reflect this close affinity to previously observed nodule sequences by including the “RF + number” designation we have utilized in previous studies of nodule Frankia (Anderson et al., 2009, 2013). All of the above‐described patterns were robust to removal of indels from the alignment using GBlocks.

Comparison of OTUs defined on the basis of sequence similarity thresholds with clade‐based OTUs illustrated a clear difference in sequence diversity between OTUs matching sequences previously found in nodules, and those only found in soils. Clade‐based nodule OTUs mostly contained sequences with >99% similarity but often included sequences with <95% similarity for non‐nodule OTUs. When all clone sequences were assigned to OTUs based on a 99% threshold, the number of OTUs defined, compared to clade‐based classification, decreased from 4 to 3 for nodule‐typical OTUs, but increased from 9 to 49 for soil‐only OTUs.

### 3.2 Distribution of *Frankia* OTUs

**FIGURE 2:**
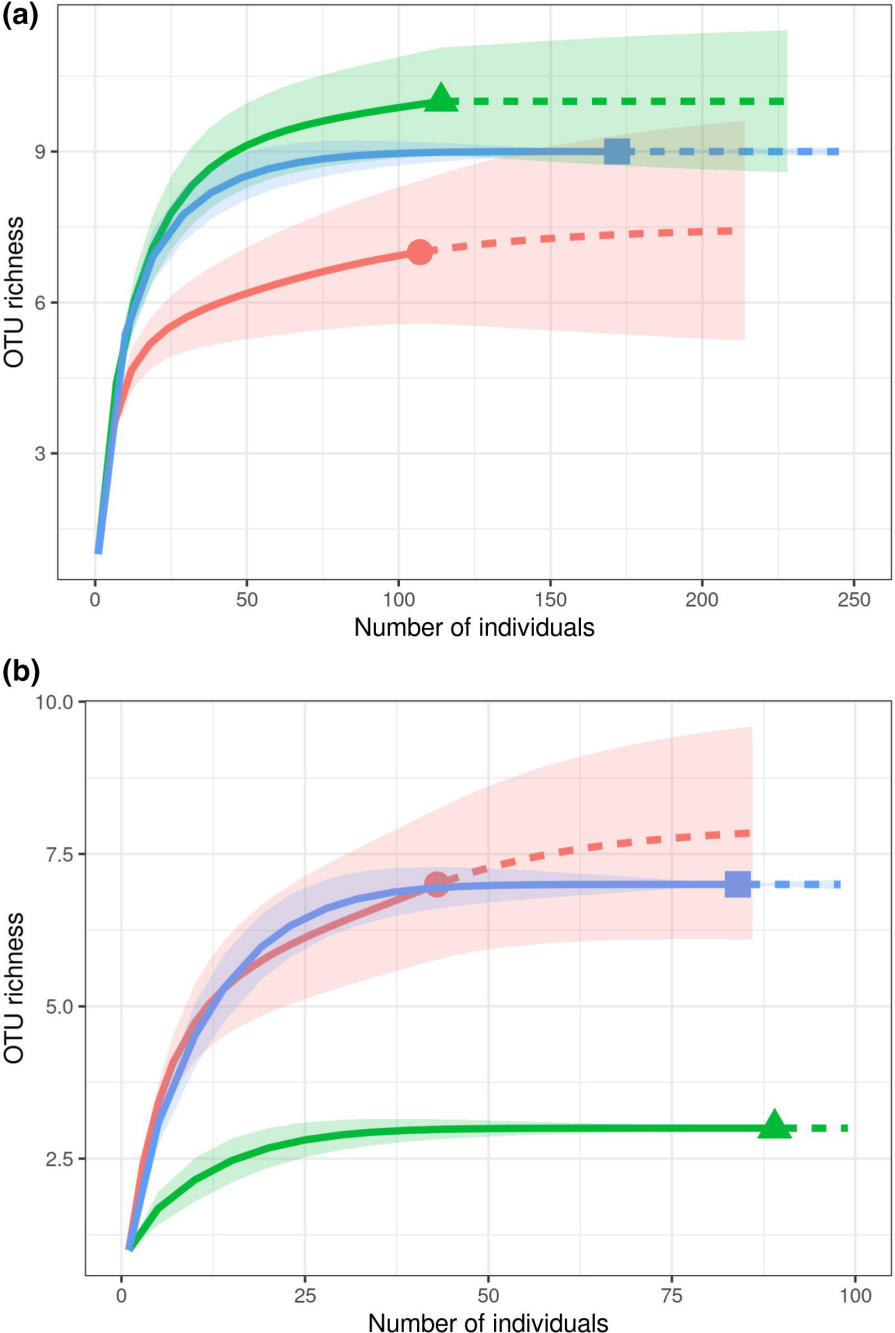
Accumulation curves of *Frankia* OTUs (sub‐clades based on rIGS locus) occurring in clones derived from soils in (a) early‐succession (*Alnus tenuifolia* stands) *A. tenuifolia* rhizospheres (red curve), late‐succession (*Picea glauca* forests) *A. tenuifolia* rhizospheres (green curve), or late‐succession areas without *A. tenuifolia* (blue curve); (b) *A. tenuifolia* rhizospheres in mid‐succession *Populus balsamifera* forests that were unfertilized (red curve), fertilized with N (NH_4_NO_3_) (green curve), or fertilized with P (P_2_O_5_) (blue curve). Shaded area around each curve represents 95% confidence intervals of estimated richness.

Rarefaction curves also illustrated the high diversity of our clone sequences. For unique sequences, curves increased linearly across all levels of sampling effort, suggesting the presence of much greater diversity in our field soils than we managed to detect (Figure S6). When OTUs were aggregated at 99% similarity, however, curves for all soils except early succession were strongly saturated, suggesting our sample was likely to have captured nearly all of the 16S‐23S IGS diversity available at that level. For clade‐based OTUs, the effect was even stronger: of the six treatments/locations we studied, all of our sample sizes were well beyond the inflection point of the OTU accumulation curve, and four of them—late‐succession alder and non‐alder, and mid‐succession N and P—appear to be well into the asymptotic portion of the curve (Figure 2). The two exceptions—early‐succession and mid‐succession unfertilized—were only modestly increasing in the extrapolation portion of the curve (Figure 2).

### 3.3 Early versus late succession

The four *Frankia* soil clades discerned in our phylogenetic analysis were non‐randomly distributed across early‐ and late‐succession soils derived from *A. tenuifolia* rhizospheres (Table 1, pseudo‐*R* ^2^ = .09, likelihood ratio (LR) *p* < .0001). Between early and late succession, proportional representation of AT clade sequences decreased significantly (Fisher's exact *p* < .0001), representation of soil clade B increased significantly (Fisher's exact *p* < .0001), and “typical *Alnus*” representation trended toward a significant increase (Fisher's exact *p* = .07).

**TABLE 1:** Frequency of *Frankia* clades occurring in clones derived from *Alnus tenuifolia* rhizosphere soils in early‐ (dense *Alnus tenuifolia* stands) or late‐succession (mature *Picea* glauca forests), or in areas lacking *A. tenuifolia* in late‐succession forests.

	Typical *Alnus*	AT clade	Clade A	Clade B	Total
Early‐succession alder soil					
Count	1	36	32	38	107
Row %	0.93	33.6	29.9	35.5	
Late‐succession alder soil					
Count	7	7	26	74	114
Row %	6.1	6.1	22.8	64.9	
Late‐succession non‐alder soil					
Count	29	6	44	93	172
Row %	16.9	3.5	25.6	54.1	
Total	37	49	102	205	393
Early vs. Late, alder	*p* = .07	*p* < .0001	ns	*p* < .0001	
Late alder vs. Non‐alder	*p* = .01	ns	ns	*p* = .09	

*Note*: *p*‐Values in bottom rows were derived from Fisher's exact test conducted for pairwise comparisons of a priori interest.

Distribution of sub‐clades was also non‐random between early‐ and late‐succession (Table 2) (pseudo‐*R* ^2^ = .17, LR *p* < .0001) soils. The largest difference occurred for the early‐succession nodule specialist RF7 (Fisher's exact *p* < .0001), which was not found in any late‐succession soils but made up >25% of the clones derived from early‐succession soils (Table 2). Significant differences also occurred for soil sub‐clades B1 (*p* = .01), B3 (*p* < .0001), and B4 (*p* = .007), which yielded 6.1%, 16.7%, and 7.0% of clone sequences from late‐succession alder soils, but were not found in early‐succession soils. Sequences related to the “typical *Alnus*” group that is dominant in nodules of *A. viridis* in our study sites were found in seven (6.1%) clones from late‐succession alder soils, but only one (0.9%) early‐succession clone. This difference trended toward significance (*p* = .07). Late‐succession soils, both within and outside of *A. tenuifolia* rhizospheres, appear to support higher OTU richness than early‐succession soil (Figure 2a).

**TABLE 2:** Frequency of *Frankia* sub‐clades occurring in clones derived from *Alnus tenuifolia* rhizosphere soils in early‐ (dense *Alnus tenuifolia* stands) or late‐succession (mature *Picea glauca* forests), or in areas lacking *A. tenuifolia* in late‐succession forests.

	Soil sequences									AT clade			Typical *Alnus*	Total
	Subclade A_1	Subclade A_2	Subclade A_3	Subclade A_4	Subclade A_5	Subclade B_1	Subclade B_2	Subclade B_3	Subclade B_4	RF1_2_3_14	RF1_2_16	RF7	RF4_8_9	
Early‐succession alder soil														
Count	12	2	0	18	0	0	38	0	0	9	0	27	1	107
Row %	11.2	1.9	0	16.8	0	0	35.5	0	0	8.4	0	25.2	0.9	
Late‐succession alder soil														
Count	8	0	0	17	1	7	40	19	8	4	3	0	7	114
Row %	7.0	0	0	14.9	0.9	6.1	35.1	16.7	7.0	3.5	2.6	0	6.1	
Late‐succession non‐alder soil														
Count	20	0	13	7	4	0	13	30	50	0	6	0	29	172
Row %	11.6	0	7.6	4.1	2.3	0	7.6	17.4	29.1	0	3.5	0	16.9	
TOTAL	40	2	13	42	5	7	91	49	58	13	9	27	37	393
Early vs. Late, alder	ns	ns	ns	ns	ns	*p* = .01	ns	*p* < .0001	*p* = .007	ns	ns	*p* < .0001	*p* = .07	
Alder vs. Non‐alder	ns	ns	*p* = .002	*p* = .002	ns	*p* = .001	*p* < .0001	ns	*p* < .0001	*p* = .02	ns	ns	*p* = .01	

*Note*: *p*‐Values in bottom rows were derived from Fisher's exact test conducted for pairwise comparisons of a priori interest.

### 3.4 Alder soils versus non‐alder soils

Differences between late‐succession soils derived from *A. tenuifolia* rhizospheres and those derived from “non‐alder” areas were much weaker than those between early‐ and late‐succession alder rhizospheres, at both the clade and sub‐clade phylogenetic scales. Among clades, the overall LR test was barely significant (*p* = .02, pseudo‐*R* ^2^ = .02), with the largest difference occurring in the proportional representation of “typical *Alnus*” sequences, which occurred in nearly 17% of non‐alder clones compared to 6% of clones derived from *A. tenuifolia* rhizosphere soil (*p* = .007) (Table 1). Differences among sub‐clades based on alder presence were stronger (LR *p* < .0001, pseudo‐*R* ^2^ = .08) than among clades (Table 2). In addition to the aforementioned difference in “typical *Alnus*” sequences, which was also significant at the sub‐clade scale (*p* = .01), the nodule‐dwelling group RF1_2_3_14 had significantly higher representation in alder than non‐alder soils (*p* = .02), although the absolute difference was only four clones versus zero. Within the soil‐only clades, several sub‐clades showed an affinity for either alder or non‐alder soils. Among the former were sub‐clades A_4 (*p* = .002), B_1 (*p* = .001), and B_2 (*p* < .0001). Among the latter, A_3 (*p* = .002) and B_4 (*p* < .0001). Both alder and non‐alder soils supported similar OTU richness (Figure 2).

### 3.5 Mid‐succession fertilization study

Fertilization treatments in the mid‐succession site also supported significantly different mixtures of *Frankia* clades (Table 3) (LR *p* < .0001, pseudo‐*R* ^2^ = .13) and sub‐clades (Table 4) (LR *p* < .0001, pseudo‐*R* ^2^ = .14). Control soils yielded a relatively even mixture of sequences belonging to the AT clade and sub‐clades unique to soil. Proportional representation of AT clade sequences was significantly higher in soils fertilized for 12 years with N (84% of clones, *p* < .0001) or 5 years with P (51% of clones, *p* = .0026) than in unfertilized control soils (23% of clones). N‐fertilized soil also contained notably lower diversity than the other two treatments, yielding less than half of the sub‐clade richness as control soils, while P‐fertilized soil yielded representatives of every sub‐clade present in control soils, albeit at different relative proportions (Figure 2, Tables 3 and 4) due primarily to the dominance of AT clade sequences in P‐fertilized soil.

**TABLE 3:** Frequency of *Frankia* clades occurring in clones derived from *Alnus tenuifolia* rhizosphere soils in mid succession (dense *Populus balsamifera* forests) that were unfertilized, fertilized with N (NH_4_NO_3_), or fertilized with P (P_2_O_5_).

	AT clade	Clade MID 1	Clade MID 2	Clade MID 3	Total
Mid‐succession control					
Count	10	5	5	23	43
Row %	23.3	11.6	11.6	53.5	
Mid‐succession N fertilized					
Count	75	0	6	8	89
Row %	84.3	0	6.7	9.0	
Mid‐succession P fertilized					
Count	43	7	14	20	84
Row %	51.1	8.3	16.7	23.8	
Total	128	12	25	51	216
Control vs. N‐fert	*p* < .0001	*p* = .003	ns	*p* < .0001	
Control vs. P‐fert	*p* = .003	ns	ns	*p* = .001	

*Note*: *p*‐Values in bottom rows were derived from Fisher's exact test conducted for pairwise comparisons of a priori interest.

**TABLE 4:** Frequency of *Frankia* sub‐clades occurring in clones derived from *Alnus tenuifolia* rhizosphere soils in mid succession (dense *Populus balsamifera* forests) that were unfertilized, fertilized with N (NH_4_NO_3_), or fertilized with P (P_2_O_5_).

	AT clade	soilMID_1	soilMID_2	soilMID_2_a	soilMID_3	soilMID_3_a	soilMID_3_a_a	Total
Mid‐succession control								
Count	10	5	4	1	14	8	1	43
Row %	23.2	11.6	9.3	2.3	32.6	18.6	2.3	
Mid‐succession N fertilized								
Count	75	0	6	0	8	0	0	89
Row %	84.3	0	6.7	0	9.0	0	0	
Mid‐succession P fertilized								
Count	43	7	6	8	9	5	6	84
Row %	51.2	8.3	7.1	9.5	10.7	6.0	7.1	
Total	128	12	16	9	31	13	7	216
Control vs. N fertilized	*p* < .0001	*p* = .003	ns	ns	*p* = .001	*p* = .0001	ns	
Control vs. P fertilized	*p* = .003	ns	ns	ns	*p* = .004	*p* = .04	ns	

*Note*: *p*‐Values in bottom rows were derived from Fisher's exact test conducted for pairwise comparisons of a priori interest.

## 4 DISCUSSION

### 4.1 Phylogenetic diversity of nodule‐ and soil‐dwelling *Frankia*

Our phylogenetic results broadly match previous studies of *Frankia* using a variety of single and multiple loci (e.g., Ghodbane‐Gtari et al., 2010; Normand et al., 1996; Nouioui et al., 2011; Pozzi, Bautista‐Guerrero, et al., 2018; Swensen & Benson, 2008). The four major host infection “Clusters” described in prior studies are evident in our tree, although their relative positions differ slightly from the most comprehensive phylogenetic characterizations (Pozzi, Bautista‐Guerrero, et al., 2018), a common result in single‐locus studies. The main ambiguity it adds to our results is whether our “Clade A” is more closely related to *Alnus*‐infective or *Elaeagnus*‐infective *Frankia*. The rest of our clone‐derived sequences fell well within previously described *Alnus*‐infective *Frankia*, and formed distinct, well‐supported clades based on whether or not they had been previously observed in nodules and, if so, which host species. Clades A and B contained sequences we have not found in nodules of either alder species native to our study area in replicated sites across three successional stages and repeated samplings spanning 6 years. Because of the rarity of alternative actinorhizal plants known to be compatible with *Alnus*‐infective *Frankia* in our study sites (Hollingsworth, 2022), we think these sequences are likely to represent free‐living, non‐symbiotic *Frankia*. In all of our unmanipulated plots, sequences from these clades were found in a clear majority of clones. This result is similar to prior surveys that also used DNA cloning to examine both nodule‐ and soil‐dwelling *Frankia* from the same field‐collected soils (Mirza, Welsh, Rieder, et al., 2009; Pokharel et al., 2011). By contrast, Kucho et al. (2019) found four clusters within the Alnus‐infective clade, only one of which occurred in soil and not nodules, and only in one clone.

Our results suggest that non‐symbiotic *Frankia* may be much more diverse than symbiotic genotypes in our sites. Our cloning method—optimized to detect sequences matching those we have found in host nodules—captured a diversity of sequences that we have never observed in nodules but clearly clustered with *Frankia* in our phylogenetic analysis. While PCR and DNA cloning are subject to well‐known biases and artifacts (e.g., Acinas et al., 2005; Chandler et al., 1997; Sipos et al., 2010; van Elsas & Boersma, 2011), we think this result is unlikely to be primarily artifactual, for three reasons. Firstly, the size of the difference is unlikely to be due to diversity‐inflating sequence artifacts alone. Depending on how we defined OTU clusters, richness of non‐symbiotic OTUs was between ~3× and ~10× greater than for symbiotic OTUs in our data (Figure S7), much greater than known rates of spurious sequence production (Acinas et al., 2005; Qiu et al., 2001; Schloss et al., 2011; Speksnijder et al., 2001). Secondly, our clones yielded sequences closely related to all of the phylogenetic clusters we observed in nodules of both alder species in our sites, suggesting sources of bias were collectively weak. Finally, we implemented several recommended precautions against known biases, including bead‐beating soils prior to DNA extraction (de Lipthay et al., 2004), use of a high‐performing DNA extraction kit (İnceoǧlu et al., 2010) supplemented with lysozyme to enhance lysis of gram‐positive bacteria (Robe et al., 2003), a balance between low enough [template DNA] to dilute PCR‐inhibiting soil compounds and high enough to minimize bias due to random priming (Chandler et al., 1997), use of degenerate primers to target a broader spectrum of template sequences, a high annealing temperature during PCR to enhance primer specificity (Sipos et al., 2010), testing of our selected cloning kit for phylogenetic bias (Taylor et al., 2007), detection and removal of sequence chimeras (which were rare), and analysis of sequence diversity by clustering into OTUs at multiple levels of similarity (Acinas et al., 2005).

Our diversity results seem to contrast with prior observations. The most dramatic difference is with Kucho et al. (2019), who found only 1 clone of 123 soil clones that belonged to a clade they did not detect in 79 nodule‐derived sequences. Pokharel et al. (2011) found restricted diversity of *Frankia* in soil compared to nodules from the same site, but only examined a single soil sample from beneath 1 of the 12 sympatric *Alnus* taxa included in their nodule sample. Mirza, Welsh, Rieder, et al. (2009) found similar diversity of soil *Frankia* and *Frankia* forming root nodules on trap plants of the “promiscuous” host *Morella pensylvanica* in six soils collected from five continents. Rodriguez et al. (2016), examining soils from three widely different ecosystems, only observed 17 unique sequences across more than 86,000 individual Illumina sequencing reads. Clustering based on 97% similarity yielded eight OTUs found in their soil samples, four of which were only found in soil, and four of which matched OTUs from reference cultures produced from host nodules.

Methodological factors could underlie the differences between these studies and ours. All four prior studies utilized nifH, a non‐neutral locus used commonly to study nodules (e.g., Higgins & Kennedy, 2012; Kennedy, Schouboe, et al., 2010; Kennedy, Weber, & Bluhm, 2010; Lipus & Kennedy, 2011; Mirza, Welsh, Rasul, et al., 2009; Mirza, Welsh, Rieder, et al., 2009; Pokharel et al., 2011; Põlme et al., 2014; Welsh, Dawson, et al., 2009; Welsh, Mirza, et al., 2009), and did not redesign their primers for the possibility of wider diversity in soils. By contrast, the non‐coding intergenic spacer we targeted is likely to vary more widely among *Frankia* due to its selective neutrality (Rocha, 2018), and the degeneracy we incorporated into our primers is likely to have targeted a broader assemblage of *Frankia*. Additionally, Mirza, Welsh, Rieder, et al. (2009) and Rodriguez et al. (2016) utilized a nested PCR protocol, reducing the amount of information available for sequence analysis from 606 bp to approximately 260 bp per sequence.

Diversity in soil has been observed in prior bioassays to be higher than in nodules of any single host species due to specificity of host and symbiont associations and wider dispersal of *Frankia* than compatible hosts (e.g., Chaia et al., 2010; McInnes et al., 2004; Mirza, Welsh, Rasul, et al., 2009). Diversity should also be higher for free‐living than symbiotic types, based on evolutionary considerations. Firstly, considering the relative evolutionary ages of plant hosts and symbiotic bacterial taxa, the free‐living lifestyle is likely to be ancestral to symbiosis (e.g., Normand et al., 1996; Sachs et al., 2014), so symbiotic lineages should tend to be nested within deeper non‐symbiotic lineages. In our data, the nesting of soil‐derived and nodule‐derived sequences relative to each other is unclear, since the branches involved had weak statistical support, and/or occurred in the portion of our tree in which placement of large clusters did not match prior studies. However, our putatively non‐symbiotic clades were clearly deeper than symbiotic ones (Figure 1, Figure S3). Secondly, soil habitats almost certainly contain a wider range of environmental conditions than the environment within host nodules, providing more opportunities for niche differentiation among free‐living than symbiotic lineages. Finally, selection imposed by hosts should exert strong purifying selection on symbionts, restricting phenotypic and genetic diversity among symbionts (Denison & Kiers, 2004), although horizontal transfer of symbiotic genes complicates this expectation (e.g., Epstein & Tiffin, 2021).

### 4.2 Host selection

Host choice theory predicts that N‐fixing plants should choose the best‐performing symbiont types from those available (Denison, 2000; Kiers & Denison, 2008; Simms & Taylor, 2002), and evolutionary constraints suggest that symbiont quality should trade‐off with the ability to survive and/or reproduce in the soil environment if the cost of maintaining both abilities is high (Roff & Fairbairn, 2007). Based on these considerations, we expected: (1) relative abundance of *Frankia* genotypes in soil to be independent from that in nodules, both within and among successional stages; (2) nodulating genotypes to be more abundant in soils beneath their host species than in areas lacking hosts; and (3) up‐regulation of nodulation via P fertilization to increase abundance of symbiotic types relative to non‐symbiotic, and down‐regulation via N fertilization to do the opposite. Our study yielded mixed results with respect to these expectations.

Our expectation that assemblages of symbiotic *Frankia* in soil and nodules would vary independently across succession was not met. In fact, the two assemblages were strikingly similar in all three successional stages we examined. Symbiont choice exerted by alder plants could produce this pattern via two opposite scenarios: (1) hosts do not exert choice, simply associating with symbiotic genotypes in proportion to their occurrence in soil; or (2) hosts do exert choice, which selectively amplifies the chosen genotypes' representation in soils. While our study cannot distinguish between these two scenarios, we think that the bulk of evidence—from both our study and prior studies of the *Alnus‐Frankia* system—favors the latter.

A great deal of circumstantial evidence supports non‐random selection of *Frankia* genotypes by alder species. A wide range of field studies has consistently reported genetic differences in *Frankia* occupying nodules collected from different alder species (Higgins & Kennedy, 2012; Lipus & Kennedy, 2011; Pozzi, Roy, et al., 2018), even when they occur in the same field site (Anderson et al., 2009; Pokharel et al., 2011; Simonet et al., 1989). Bioassay studies of field soils have reported differences in nodulating *Frankia* between different *Alnus* species grown in the same soil (Ben Tekaya et al., 2018; Lipus & Kennedy, 2011), and cross‐inoculation studies using crushed nodules or isolates have indicated differences in compatibility between host species and specific *Frankia* strains (Du & Baker, 1992; Markham, 2008; Prat, 1989). Recent microcosm studies have provided more direct evidence. Ben Tekaya et al. (2018) used qPCR and Illumina sequencing to compare the relative proportions of *Frankia* genotypes in soils with those in associated nodules of three European and North American *Alnus* species. Nodule assemblages differed strongly among the two alder species that formed nodules in their soils and also varied independently from soil assemblages. Importantly, soil assemblages were characterized after a short enough time (7 months) that nodule senescence‐driven feedback was unlikely to have contributed strongly to their result. Vemulapally, Guerra, and Hahn (2022) conducted a similar study but included one additional North American alder species and a treatment in which equal mixtures of genetically distinct *Frankia* isolates were also inoculated into field soils. Both inoculated and indigenous genotypes grew to different densities in soils over a 3‐month timeframe, but similar relative proportions of genotypes occurred across different host species. By contrast, nodules contained different mixtures of bacterial genotypes among alder species, and again the relative proportions in nodules were independent from those in associated soils. The selective ability of alder is also supported by the field study of Mirza, Welsh, Rieder, et al. (2009), in which alder nodules were found to contain high frequencies of some genotypes that were undetectable in soil clones.

Selection of specific genotypes by host plants has strong potential to feed back to soil assemblages of N‐fixing bacteria (West et al., 2002). Density of bacterial cells in nodules is many orders of magnitude greater than in surrounding soils (Denison, 2000; Denison & Kiers, 2004), and nodule‐dwelling bacteria maintain connections with soil assemblages via extra‐nodular extensions of hyphae (Diem et al., 1982) or infection threads (Denison, 2000), or release of viable cells (Denison, 2000) or spores (Pozzi et al., 2015) during nodule senescence. Rhizobial densities have been observed to increase during nodule senescence (Brockwell et al., 1987; Denison & Kiers, 2011), and nodulating capacities of soil‐dwelling *Frankia* often increase near host plants (Chaia et al., 2010), providing circumstantial evidence of the importance of nodule feedback in maintaining symbiotic populations.

In our study, enhancement of symbiotic assemblages by hosts is suggested by two specific results. First, the proportion of total clones yielding symbiotic genotypes found in rhizosphere soils decreases monotonically from early (0.346), to mid (0.233), to late succession (0.061) (Table 1). This pattern parallels both the density of host plants and the density of nodules within host rhizospheres across succession, indicating a correspondence between the availability of host interactions and the relative representation of symbiotic genotypes in soil assemblages. Second, this trend continues in the frequency of AT clade genotypes, particularly compared with non‐symbiotic ones, in host rhizospheres (proportion of symbiotic genotypes = 0.061) versus non‐alder soils (0.035) in late succession (Table 1). Interestingly, the highest frequency of typical *Alnus* genotypes observed in our study also occurred in late‐succession non‐alder soil. We have only rarely observed this group in *A. tenuifolia* nodules, which raises the intriguing possibility that non‐host‐specific symbiotic types may be inhibited in rhizospheres of this alder species, in addition to enhancement of specific nodule‐forming genotypes. This speculation, of course, will require further evidence to confirm.

Our most surprising finding, compared with a priori expectations, occurred in response to long‐term fertilization in the mid‐succession site. Addition of N and P has well‐known effects on nodule formation by alders that are opposite in direction (Wall & Berry, 2008). In our study, nodule formation differed among treatments in the expected direction: P significantly increased the number of nodules per unit area (4631 ± 1051 clusters/m^2^), and N significantly decreased it (346 ± 159 clusters/m^2^), compared with unfertilized control plots (911 ± 199 clusters/m^2^) (Ruess et al., 2013). Based on the relative differences in opportunities for host interactions and soil feedback provided by these two responses to fertilization, we expected P to enhance host‐selected genotypes in host‐associated soils, particularly compared with non‐symbiotic types, and N to decrease this proportion. Consistent with the former expectation, the proportion of total clones yielding symbiotic genotypes was nearly double in soils under long‐term P fertilization (0.512) compared to the value in control soils (0.233). Contrary to expectation, however, it reached its highest value (0.843) under N fertilization (Table 1).

In a system as complex as soil, the number of hypotheses we could propose to explain this unexpected result is nearly infinite. A useful distinction between kinds of explanation is the dichotomy between mechanisms mediated by hosts and those that are not. The size of the fertilization effect, together with the fact that symbiotic types were disproportionately enhanced, would seem to implicate host‐mediated mechanisms. Two non‐exclusive processes seem most likely to be involved. First, addition of N may have produced an influx of nodule‐dwelling *Frankia* into surrounding soil by triggering large‐scale nodule senescence. There is some evidence that this may have occurred: live nodule biomass was lower in N‐fertilized (21.0 ± 3.4 g/m^2^) than control plots (33.5 ± 5.9 clusters/m^2^), and dead biomass was higher under N fertilization (6.7 ± 1.8 g/m^2^) than in control (4.3 ± 1.2 g/m^2^) plots (Ruess et al., 2013). Although neither difference was statistically significant, it is plausible that they could have amounted to a difference in input of nodule‐dwelling genotypes. The latter difference could have amplified the difference in nodule biomass changes, particularly in response to our second hypothetical process: N‐enhanced growth of host plants. Although we did not measure any growth‐related variables in our host plants, mineral N is known to increase growth in seedlings of several alder species under laboratory conditions, even when the seedlings are symbiotically fixing N (Ingestad, 1980; Radwan, 1987; Stewart & Bond, 1961). Even as nodulation continued to decrease over subsequent years, any initial influx of symbiotic genotypes from senescent nodules could have been further enhanced in soil by, for example, plant growth‐stimulated production of leaf litter (as observed by Mirza et al., 2007; Mirza, Welsh, & Hahn, 2009) and/or unidentified host‐associated compounds (as observed by Mirza et al., 2007; Samant et al., 2015, 2016), both of which have been observed to preferentially enhance soil‐dwelling populations of specific symbiotic *Frankia* genotypes. Obviously, this scenario is speculative and will require further study to confirm. Overall, our results indicate that the transition between soil‐ and nodule‐dwelling states is likely to be a significant contributor to the eco‐evolutionary complexity that increasingly appears to define these symbiotic interactions.

### 4.3 Study limitations

In this study, we found strong differences in soil‐dwelling *Frankia* among successional stages and fertilization treatments that largely parallel the pattern of differences we have observed in *Frankia*‐inhabiting nodules of the host plant *Alnus incana* ssp. *tenuifolia*. However, the strength of our conclusions in the present study is subject to several limitations of our design. First, because we only included one representative site for each successional stage, we cannot confirm that the pattern we have observed in nodules—in which variation among stages exceeds variation among sites representing the same stage—also holds for soil assemblages. Second, because we pooled soil samples within each stage/treatment combination, we also cannot estimate variation in soil‐dwelling *Frankia* within these sampling units. Finally, our mid‐succession samples were collected a year after our early‐ and late‐succession ones. For the latter two points, our design relies on the assumption that the robust patterns we have observed in nodules—in which within‐site variation is small compared to among‐stage, and that assemblage structure is stable interannually—also apply to soil‐dwelling *Frankia*. However, this assumption awaits rigorous confirmation.

## AUTHOR CONTRIBUTIONS

**M. D. Anderson:** Conceptualization (equal); data curation (lead); formal analysis (lead); investigation (lead); methodology (equal); project administration (equal); writing – original draft (lead); writing – review and editing (lead). **D. L. Taylor:** Conceptualization (equal); methodology (equal); project administration (equal); supervision (equal); writing – review and editing (supporting). **R. W. Ruess:** Conceptualization (equal); funding acquisition (lead); project administration (equal); supervision (equal); writing – review and editing (supporting). **K. Olson:** Investigation (supporting); methodology (supporting).

## FUNDING INFORMATION

We are indebted to NSF's Ecosystem Studies Program for their confidence in our ideas and for funding DEB‐0641033 to R.W. Ruess and team. Additional funding was provided by the Bonanza Creek LTER Program, which is jointly funded by NSF (DEB 0620579) and the USDA Forest Service, Pacific Northwest Research Station (PNW01‐JV11261952‐231).

## CONFLICT OF INTEREST STATEMENT

The authors have no conflicts of interest to declare.

## Supporting information


Appendix S1.

## Data Availability

The data that support the findings of this study are openly available on GenBank at https://www.ncbi.nlm.nih.gov/genbank, accession numbers OR033811–OR034096 (nodules and mid‐succession‐only clones) and OR033183–OR033810 (nodules, cross‐succession, and alder vs. non‐alder clones).
