# Supplementary material for: Composition of soil Frankia assemblages across ecological drivers parallels that of nodule assemblages in Alnus incana ssp. tenuifolia in interior Alaska
Source: Ecol Evol. 2024 Jul 8;14(7):e11458. doi: 10.1002/ece3.11458 (PMC11229434; doi:10.1002/ece3.11458)
Supplement: Supplementary file 1 — Appendix S1. [file ECE3-14-e11458-s001.zip › ece311458-sup-0001-AppendixS1.docx]

Anderson et al. 2023: Supplemental Materials


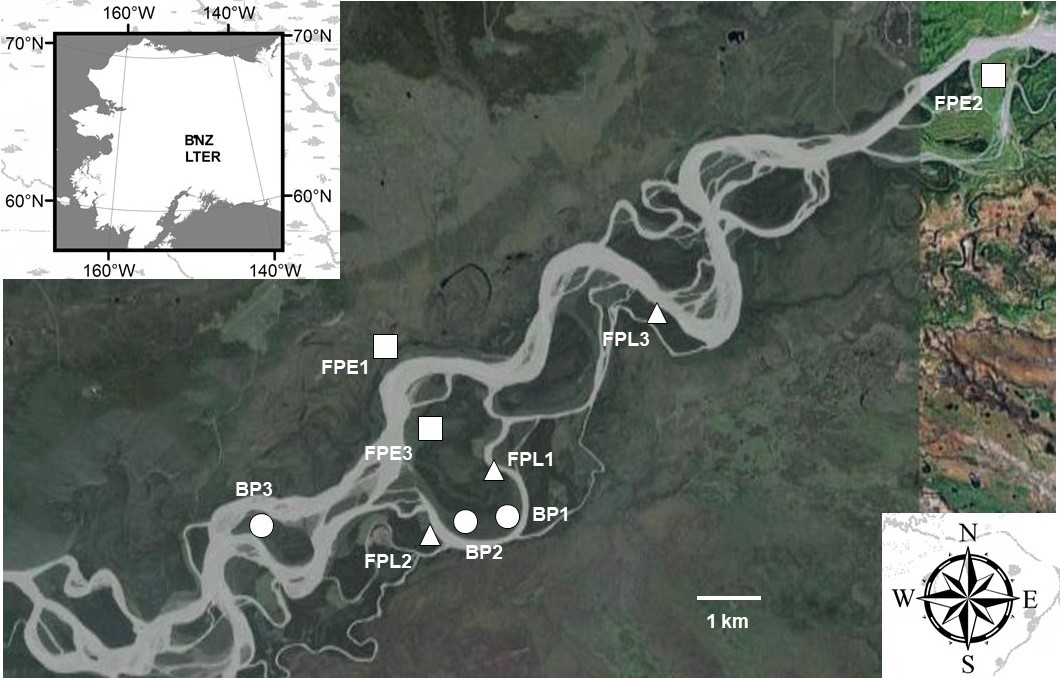


Figure S1. Map of sites included in the study. Study sites were located in the Bonanza Creek (BNZ) Experimental Forest, a National Science Foundation funded Long-Term Ecological Research (LTER) site. Top left inset shows location of the BNZ-LTER within Alaska. Map includes all sites examined in our wider research program: three early succession sites (squares), three mid-succession sites (circles) and three late-succession sites (triangles). Sites in the present study are a subset of these previously studied sites: one early succession (FPE3), one mid-succession (BP1), and one late succession (FPL2).


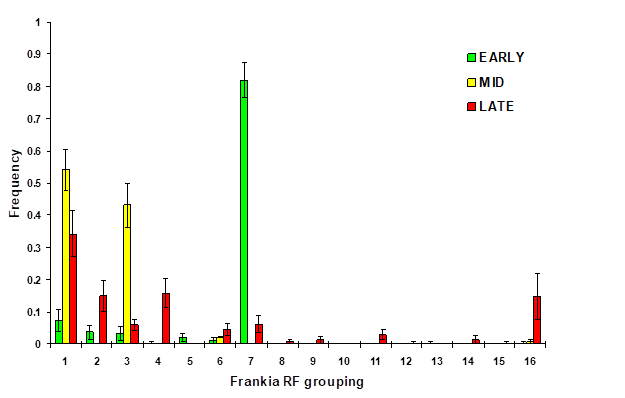


Figure S2. Mean frequencies (± 1 standard error of the mean) of previously observed *Frankia* genotypes in nodules of *Alnus tenuifolia* collected from three early, mid, and late-succession sites that include those used in the present study. Early succession sites were sampled in 2002, 2005 and 2008. Late succession sites were sampled in 2002 and 2005. Mid succession sites were sampled in 2008 only. Means and standard errors were calculated across all three replicate sites and all sampling years.


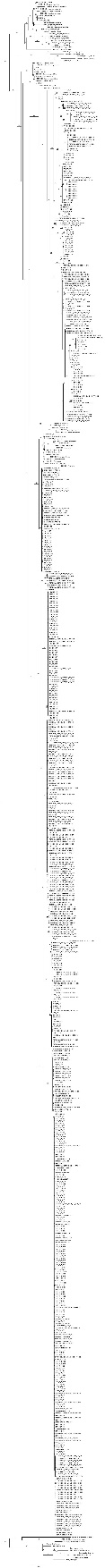


Figure S3. Fully expanded phylogeny of early and late succession soil clones represented. Collapsed version is manuscript Figure 1. A high resolution version of this tree is provided as a separate supplemental file.


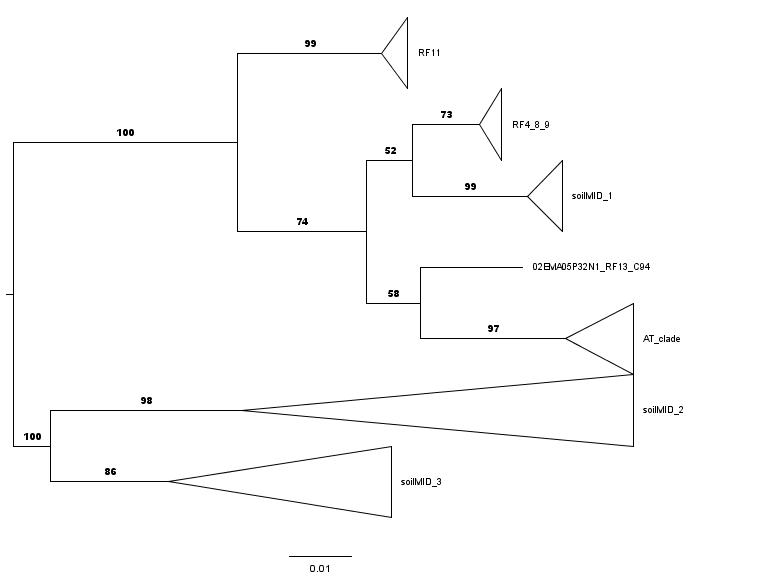


Figure S4. Phylogenetic tree of mid-succession soil clades.


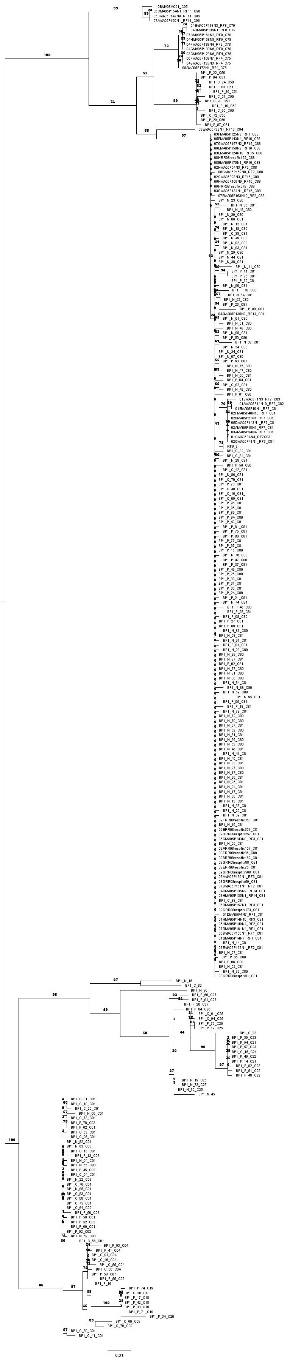


Figure S5. Fully expanded tree of mid-succession soil clones. A high resolution version of this tree is provided as a separate supplemental file.

S6.a.


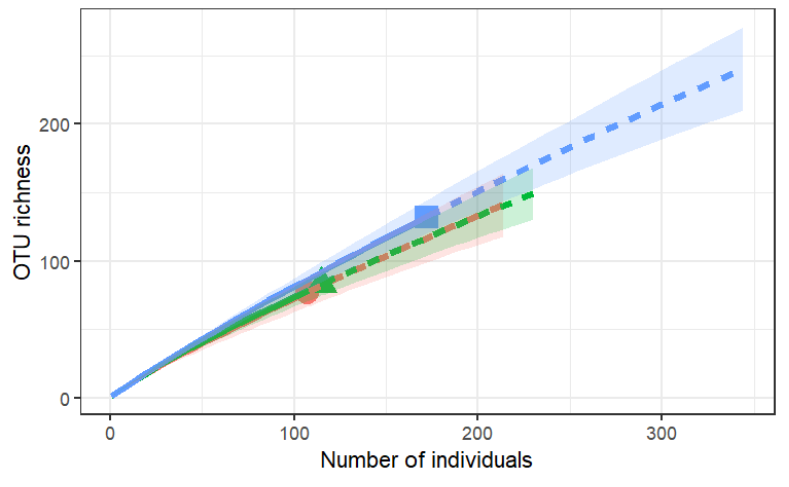


S6.b.


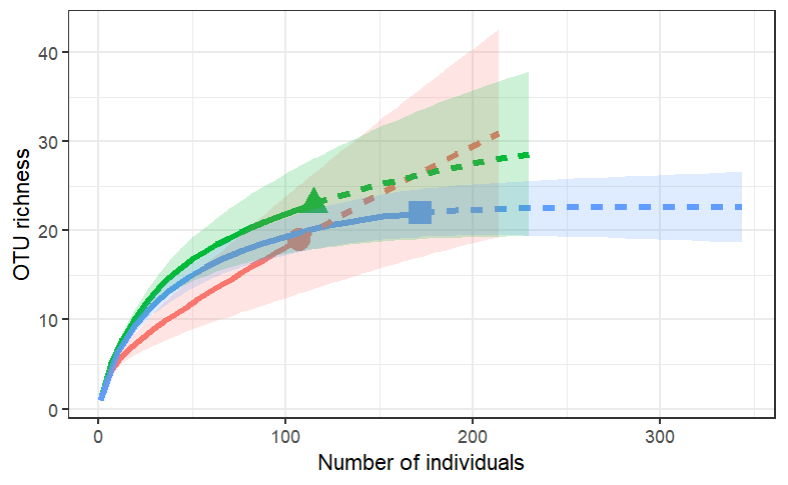


S6.c.


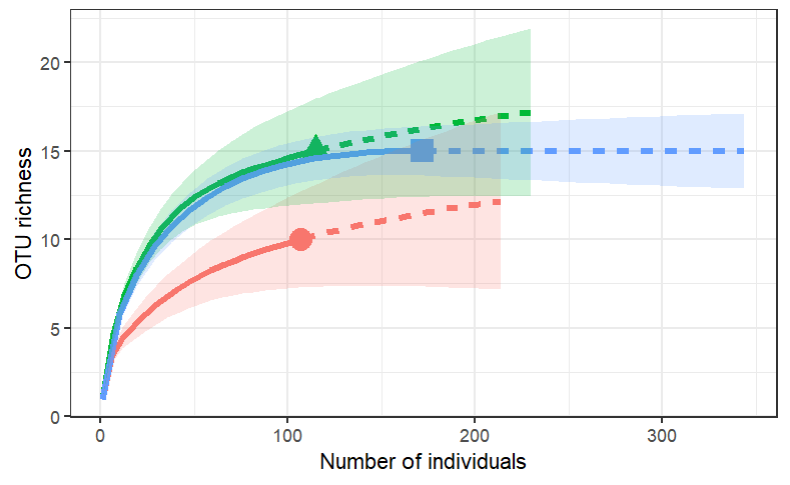


Figure S6. Accumulation curves for OTUs defined at different similarity thresholds: unique sequences (a), 1% similarity (b), and 3% similarity (c). Comparison curves represent early succession (red), late succession alder rhizospheres (green), and late succession soils with no alder present (blue).

S7.a.


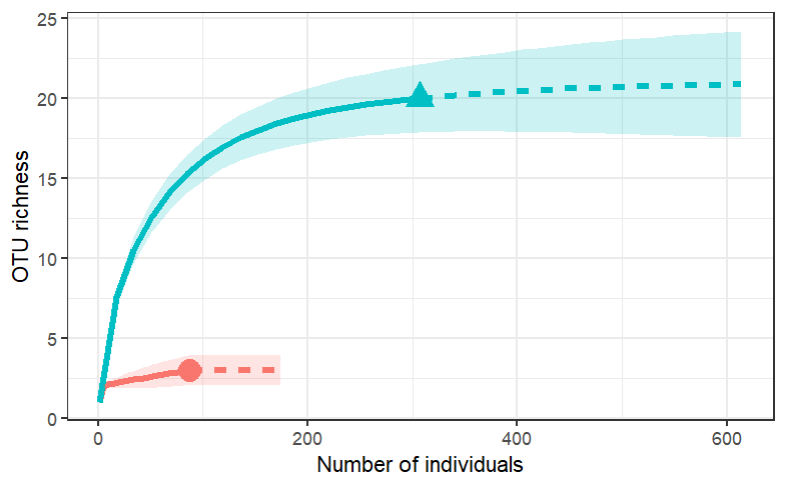


S7.b.


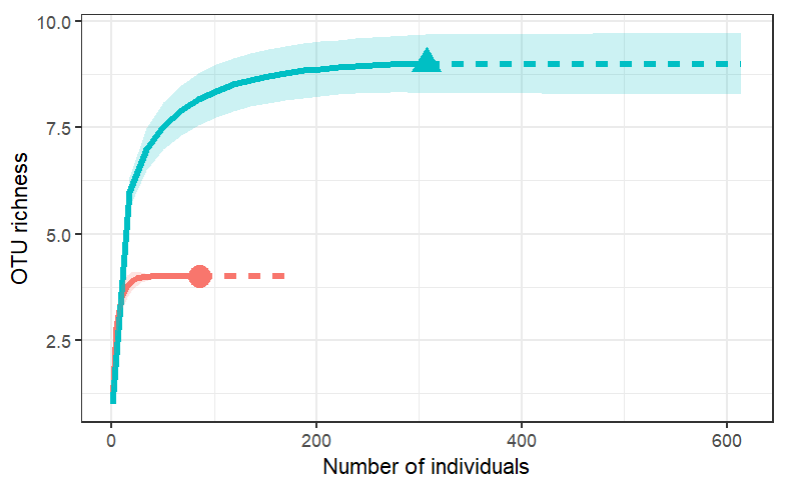


Figure S7. Comparison of accumulation curves between symbiotic (red) and non-symbiotic (blue) genotypes for OTUs defined at 3% similarity (a) or based on phylogenetic sub-clades (b).
