# Supplementary material for: Composition of soil Frankia assemblages across ecological drivers parallels that of nodule assemblages in Alnus incana ssp. tenuifolia in interior Alaska
Source: Ecol Evol. 2024 Jul 8;14(7):e11458. doi: 10.1002/ece3.11458 (PMC11229434; doi:10.1002/ece3.11458)

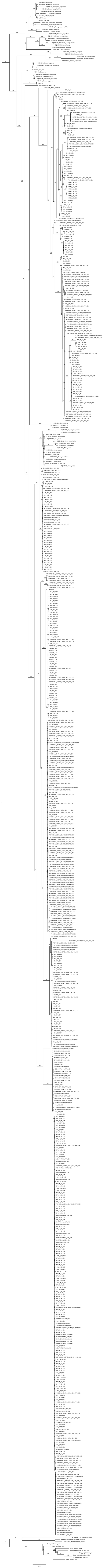

Supplement: Supplementary file 1 — Appendix S1. [file Figure S3 high resolution.pdf › Figure S3 high resolution.pdf]
